# Prevalence and associated factors of postpartum depression symptoms among mothers in Mogadishu, Somalia: A Hospital‐based Cross‐sectional Study

**DOI:** 10.1016/j.xjmad.2025.100157

**Published:** 2025-11-14

**Authors:** Rahma Yusuf Haji Mohamud, Nur Adam Mohamed, Yusuf Abdirisak Mohamed, Khadija Yusuf Ali, Amal Nor Ali, Said Mohamed Mohamud, Serpil Doğan, Nazan Karahan, Said Abdirahman Ahmed, Alia Ismail Hashi, Zerife Orhan, Mohamed Yaqub Hassan

**Affiliations:** a Department of Nursing, Mogadishu Somali-Turkiye Recep Tayyip Erdogan Research and Training Hospital, Mogadishu, Somalia; b Jazeera university faculty of health sciences, Mogadishu, Somalia; c Department of Psychiatry, Mogadishu Somali-Turkiye Recep Tayyip Erdogan Training and Research Hospital, Mogadishu, Somalia; d Faculty of Medicine and Surgery, Somali National University, Mogadishu, Somalia; e Department of Obstetrics and Gynecology, Mogadishu Somali-Turkiye Recep Tayyip Erdogan Research and Training Hospital, Mogadishu, Somalia; f Department of Pulmonology, Mogadishu Somali-Turkiye Recep Tayyip Erdogan Research and Training Hospital, Mogadishu, Somalia; g Department of Medical Microbiology, Mogadishu Somali-Turkiye Recep Tayyip Erdogan Research and Training Hospital, Mogadishu, Somalia; h Sağlık Bilimleri Üniversitesi Gülhane Sağlık Bilimleri Fakültesi Ebelik Bölümü, Istanbul, Turkey; i Department of Cardiology, Mogadishu Somali Turkish Training and Research Hospital, Mogadishu, Somalia; j Department of Nursing, University of Health Sciences, Mogadishu, Somalia; k Kahramanmaraş Sütçü İmam University, Vocational School of Health Services, Kahramanmaraş, Turkey; l Department Pediatrics, Mogadishu Somali-Turkiye Recep Tayyip Erdogan Research and Training Hospital, Mogadishu, Somalia

**Keywords:** Postpartum depression, Postpartum mothers, Associated factors, Mogadishu, Somalia

## Abstract

**Introduction:**

Postpartum depression (PPD) is a major public health concern, affecting an estimated 10–15 % of women worldwide, with higher rates consistently reported in low- and middle-income countries. This study sought to estimate the prevalence of PPD symptoms and identify associated risk factors among postpartum mothers in Mogadishu, Somalia.

**Methods:**

A hospital-based cross-sectional study was conducted at Erdogan Hospital, Mogadishu, between November 1 and December 31, 2023. Using systematic sampling, 271 postpartum mothers were recruited. Data were collected through structured questionnaires assessing sociodemographic, obstetric, and infant-related characteristics. The Edinburgh Postnatal Depression Scale (EPDS) was used to screen for PPD symptoms, while the Maternity Social Support Scale (MSSS) measured perceived social support. Bivariate and multivariable logistic regression analyses were performed to identify factors associated with PPD., and results were reported as adjusted odds ratios (AOR) with 95 % confidence intervals (CIs). Statistical significance was set at p < 0.05.

**Results:**

Of the 271 participants, 26.6 % (95 % CI: 21.4 %–32.2 %) screened positive for PPD symptoms. Significant variables associated with PPD symptoms included stressful life events (AOR = 14.46; 95 % CI: 5.75–36.34), history of depression (AOR = 3.11; 95 % CI: 1.36–7.08), infant sleep difficulties (AOR = 2.53; 95 % CI: 1.27–5.05), pregnancy complications (AOR = 2.55; 95 % CI: 1.12–5.82), and low (AOR = 12.72; 95 % CI: 3.77–42.90) or moderate (AOR = 2.67; 95 % CI: 1.28–5.60) levels of social support.

**Conclusions:**

This study demonstrates that more than one in four mothers in Mogadishu experience symptoms of postpartum depression. The findings underscore the need for healthcare professionals to prioritize routine screening for psychological distress, closely monitor women at higher risk, provide timely interventions, and strengthen social support systems. Integrating targeted psychoeducational programs into perinatal care and developing culturally sensitive mental health services could play a critical role in reducing the burden of PPD in Somalia.

## Highlights

• Over one in four postpartum mothers in Mogadishu screened positive for depression symptoms.
• Stressful life events were strongly associated with postpartum depression symptoms.
• A prior history of depression was significantly associated with postpartum depression symptoms.
• Pregnancy complications and infant sleep problems were associated with postpartum depression symptoms.
• Strong social support showed a protective association against postpartum depression symptoms.

## Introduction

Postpartum depression (PPD) is one of the most prevalent mental health disorders affecting women worldwide, with an estimated 10–15 % of mothers experiencing it after childbirth [1], [2]. According to the Diagnostic and Statistical Manual of Mental Disorders, Fifth Edition (DSM-5), PPD is defined as a major depressive episode with onset during pregnancy or within four weeks following delivery [3] However, both clinical and research settings commonly extend this timeframe to include depressive symptoms arising at any point within the first year postpartum [4]. Typical symptoms include persistent sadness, loss of interest or pleasure, changes in sleep and appetite, fatigue, excessive guilt, impaired concentration, irritability, frequent crying, and—in severe cases—thoughts of self-harm or harm to the infant [5]. These symptoms can significantly impair maternal functioning and caregiving capacity.

PPD poses a serious public health challenge, disproportionately affecting an estimated 19 % of women in low- and middle-income countries [6]. While its exact etiology remains unclear, a combination of biological, psychological, and social factors has been implicated. These include genetic vulnerability, hormonal shifts during and after pregnancy, birth-related trauma, and various psychosocial and demographic stressors [6], [7], [8]. Research from Southwest Ethiopia has identified maternal age, unintended pregnancy, previous depression, chronic illness, limited social support, domestic violence, infant loss, infant sleep problems, marital instability, and substance use as key determinants of PPD [9], [10]. Similarly, a recent Kenyan study highlighted partner conflict and financial hardship as significant contributors [11]. Furthermore, a systematic review and meta-analysis identified factors such as gestational diabetes mellitus, antenatal depression, having a male infant, and the use of epidural anesthesia during delivery [12].

Risk factors for PPD differ across cultural and socioeconomic contexts, shaped by childbirth practices, gender norms, stigma, and unequal access to mental-health care. These contextual pressures may heighten vulnerability in resource-limited settings such as Somalia. Recognizing these nuances is critical for designing context-appropriate prevention, targeted screening, and clear referral pathways for women at elevated risk [6]. In countries with limited healthcare resources such as Somalia, identifying factors associated with PPD is particularly critical due to the scarcity of specialized mental health services. Early recognition of high-risk mothers enables targeted screening, timely diagnosis, and appropriate intervention, all of which can alleviate depressive symptoms, improve maternal and child health outcomes, and strengthen mother–infant bonding [13].

Despite high fertility and substantial maternal health challenges in Mogadishu [14], context-specific evidence on postpartum depression remains limited, constraining service planning and policy. By quantifying local prevalence and modifiable risk factors, this study provides actionable inputs for maternal health services: integrating routine EPDS screening into postnatal and immunization contacts; developing culturally adapted, partner-inclusive psychoeducation and social-support interventions; establishing clear referral pathways to mental-health care; and guiding targeted staff training and resource allocation in high-risk clinics. These findings can directly inform municipal and national policy decisions to embed PPD screening and stepped-care within maternal and child health programs.

## Methods

### Study design and setting

A hospital-based cross-sectional study was conducted among postpartum mothers attending the Expanded Program on Immunization (EPI) clinic at the Mogadishu Somali-Turkiye Training and Research Hospital in Mogadishu, Somalia, from November 1 to December 31, 2023. Each participant completed both sections of the self-administered questionnaire during a single session on the same day, immediately after their child’s immunization visit, ensuring temporal consistency across all responses.

Erdogan Hospital is a public, teaching, and tertiary referral hospital initially established in the 1960s. The hospital operated continuously until the onset of civil war and government collapse in the early 1990s, subsequently ceasing operations. Following restoration efforts by the Somali and Turkish governments, the hospital reopened in January 2015 and currently serves as a major healthcare provider in the region.

### Sample size and sampling procedure

The sample size was calculated using the single-population proportion formula, with a 95 % confidence level (Z = 1.96), an expected prevalence of 59.9 % from a prior Mogadishu study [15], and a 5 % margin of error. This yielded an initial estimate of 369. After applying finite population correction for a clinic population of 730, the required sample size was reduced to 246. Allowing for a 10 % non-response rate, the final target sample was set at 271. Systematic sampling was then employed: the interval was obtained by dividing the total number of eligible postpartum mothers (730) by the target sample (271), resulting in approximately 2.7. This was rounded to three, meaning every third mother was selected. To ensure randomness, a lottery was used to choose the starting point from among the first three mothers, and the number two was drawn; thus, the second mother was the first participant, followed by every third eligible mother thereafter (2nd, 5th, 8th, 11th, etc.) until the target of 271 was reached. This approach ensured both randomization at the outset and even distribution across the sampling frame.

### Inclusion and exclusion criteria

Eligible participants were postpartum mothers aged 18 years or older who had delivered within the six months preceding data collection and attended the hospital's EPI clinic during the study period. Mothers who were severely ill or had hearing impairments were excluded from participation.

### Variables of study

#### Dependent variable

Postpartum depressive symptoms, assessed using the Edinburgh Postnatal Depression Scale (EPDS). Participants scoring ≥ 11 were considered to have probable postpartum depressive symptoms, while scores < 11 indicated the absence of these symptoms.

#### Independent variables

Socio-demographic factors: age, marital status, education, occupational status, monthly household income (in USD), presence of chronic medical conditions, stressful life events, and history of depression. Obstetric and infant-related factors: breastfeeding status, breastfeeding difficulties, pregnancy intention (planned or unplanned), outcome of the previous pregnancy, parity, mode of delivery, history of abortion, place of delivery, newborn illness, infant hospitalization status, infant gender, and pregnancy complications. Social support: categorized as low, medium, or high based on the maternity social support scale (MSSS).

### Data collection procedures and tools

Data were collected through self-administered questionnaires, divided into two main sections. The first section comprised structured, closed-ended questions covering socio-demographic characteristics, obstetric histories, and infant-related variables. This structured questionnaire was developed based on literature review [16].

The second section included two validated instruments: The EPDS is a 10-item, validated screening instrument designed to detect depressive symptoms in postpartum women in both community and clinical settings. Each item assesses the severity of symptoms experienced over the past week on a 4- point Likert scale ranging from 0 to 3, with total scores ranging from 0 to 30. A cut-off score of ≥ 11 indicated probable postpartum depressive symptoms, consistent with prior validation studies. It should be noted that the EPDS is a screening tool rather than a diagnostic instrument; it identifies individuals who warrant further psychiatric evaluation [17], [18].

Maternity Social Support Scale (MSSS): Perceived social support among postpartum mothers was assessed using the MSSS, a validated 6-item scale originally developed by Webster et al. [19]. The scale evaluates support from friends, family, and spouse/partner, as well as aspects of partner conflict, control, and affection. Items are rated on a 5-point Likert scale, yielding total scores from 6 to 30. According to the original validation study, scores < 19 indicate low support, 19–24 indicate medium support, and > 24 indicate high support.

### Data management and statistical analysis

Collected data were initially entered and cleaned using Microsoft Excel before being transferred into the Statistical Package for the Social Sciences (SPSS), version 26 (Armonk, NY: IBM Corp.). Data cleaning involved checking for completeness, detecting and removing duplicate or inconsistent entries, and correcting coding errors to ensure data accuracy and integrity.

Descriptive statistics were computed to summarize participant characteristics across socio-demographic, obstetric, infant-related, and social support domains. Frequencies and percentages were used to describe all categorical variables. The prevalence of postpartum depressive symptoms was estimated, and the corresponding 95 % confidence intervals were derived using the Clopper–Pearson exact method [20]. To identify factors associated with postpartum depressive symptoms, both bivariate and multivariable logistic regression analyses were performed. Variables with a p-value ≤ 0.25 in bivariate analyses were considered for inclusion in the multivariable model, following the recommendations of Bursac et al. [21], to ensure that potential confounders were not excluded prematurely. Two separate logistic regression models were constructed: one including socio-demographic and social-support variables, and another including obstetric and infant-related variables. This approach was chosen to maintain an adequate events-per-independent-variable (EPV) ratio, minimize multicollinearity, and clarify the domain-specific contribution of variables associated with postpartum depressive symptoms. To assess model validity, the Hosmer–Lemeshow test was employed to evaluate goodness-of-fit [22], with the corresponding chi-square values and p-values reported alongside each regression table. The results indicated adequate fit for the models (e.g., Table 3: χ² = 10.16, p = 0.18; Table 4: χ² = 11.30, p = 0.13). Variance inflation factors (VIF) and correlation matrices were also examined to detect multicollinearity, and all variables demonstrated acceptable VIF scores (<2.5), confirming the absence of problematic collinearity. Associations were reported as adjusted odds ratios (AORs) with corresponding 95 % confidence intervals (CIs), and statistical significance was defined as p < 0.05.

### Ethical approval

Ethical approval was obtained from the Institutional Review Board of Mogadishu Somali-Turkiye Training and Research Hospital. Prior to enrollment, participants received comprehensive information about the study objectives, and written informed consent was obtained voluntarily. They were assured of their right to withdraw at any stage without penalty. Confidentiality was strictly maintained throughout data collection and analysis, with no personal identifiers collected or reported. Participants who scored ≥ 11 on the EPDS, suggesting possible postpartum depression symptoms, were discreetly informed of their results and referred to the hospital’s mental health unit for further assessment and care. When necessary, trained data collectors also provided immediate emotional support and psychoeducation.

## Results

### Socio-demographic characteristics

A total of 271 postpartum mothers participated in the study, yielding a 100 % response rate. The majority were aged 21–30 years (67.2 %), married (90.8 %), and unemployed (77.1 %). Over half of the participants reported a monthly household income of ≤ 500 USD (53.5 %). Most respondents had no chronic illness (88.9 %), no prior history of depression (79.7 %), and no recent exposure to stressful life events (84.9 %) (Table 1).

**Table 1:** Socio-demographic and social support characteristics of postpartum mothers in Mogadishu, Somalia, 2024 (n = 271).

**Variable**	**Category**	**Frequency**	**Percentage**
Age	20 and below	13	4.8
	21–30	182	67.2
	31 and above	76	28
Marital status	Married	246	90.8
	Widowed	15	5.5
	Divorced	10	3.7
Education status	Non-formal education	66	24.4
	Primary school	32	11.8
	Secondary school	67	24.7
	Degree and above	106	39.1
Occupation status	Employed	62	22.9
	Unemployed	209	77.1
Monthly income	≤ 500 USD	145	53.5
	501–1000 USD	107	39.5
	> 1000 USD	19	7.0
Having chronic illness	Yes	30	11.1
	No	241	88.9
Stressful life events	Yes	41	15.1
	No	230	84.9
History of depression	Yes	55	20.3
	No	216	79.7
Perceived social support	Low	20	7.4
	Medium	111	40.9
	High	140	51.7

### Obstetric and infant-related characteristics

Nearly half of the participants (44.6 %) reported having 2–4 pregnancies, while approximately 10 % experienced complications during pregnancy. Non-exclusive breastfeeding was practiced by the majority (54.6 %), and breastfeeding difficulties were reported by 21.8 %. In terms of delivery, about one-third of mothers (32.1 %) gave birth outside formal healthcare facilities, and more than one-fifth (21.8 %) reported hospitalization of their newborns (Table 2).

**Table 2:** Obstetric, baby, and social support factors of postpartum mothers in Mogadishu, Somalia, 2024 (n = 271).

**Variable**	**Category**	**Frequency**	**Percentage**
Number of pregnancies	1	52	19.2
	2–4	121	44.6
	Above 4	98	36.2
Breastfeeding status	Exclusive	123	45.4
	Non-exclusive	148	54.6
Breastfeeding problems	Yes	59	21.8
	No	212	78.2
Planned pregnancy	Yes	145	53.5
	No	126	46.5
Complications during pregnancy	Yes	28	10.3
	No	243	89.7
Mode of delivery	Spontaneous vaginal	140	51.7
	Cesarean section	131	48.3
Place of delivery	Healthcare center	184	67.9
	Non-healthcare center	87	32.1
History of abortion	Yes	120	44.3
	No	151	55.7
Gestational age of the last pregnancy	Pre-term	19	7.0
	Full-term	220	81.2
	Post-term	32	11.8
Gender of the baby	Male	113	41.7
	Female	158	58.3
Problems with baby sleeping	Yes	44	16.2
	No	227	83.8
Complications at childbirth	Yes	13	4.8
	No	258	95.2
Newborn illness	Yes	31	11.4
	No	240	88.6
Baby hospitalized	Yes	59	21.8
	No	212	78.2

### Prevalence of postpartum depressive symptoms

**Fig. 1:**
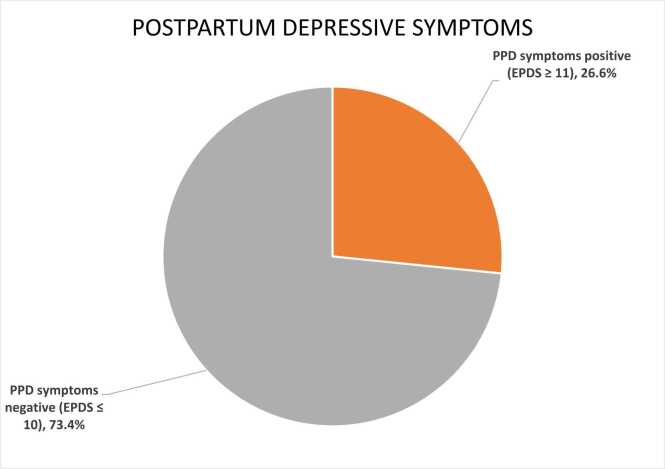
Prevalence of postpartum depressive symptoms among postpartum mothers in Mogadishu, Somalia.

The overall prevalence of probable postpartum depression symptoms, defined as an EPDS score ≥ 11, was 26.6 % (95 % CI: 21.4 %–32.2 %). This result indicates that more than one in four postpartum mothers experienced clinically significant depressive symptoms (Fig. 1).

### Socio-demographic and social support factors associated with PPD symptoms

In the bivariate logistic regression analyses, education status, presence of chronic illness, stressful life events, history of depression, and low or medium social support were significantly associated with postpartum depressive symptoms. However, after adjusting for potential confounders in the multivariable model, only stressful life events, history of depression, and low or medium social support remained significant independent correlates of postpartum depressive symptoms (p < 0.05).

Specifically, postpartum mothers who experienced stressful life events had markedly higher odds of developing depressive symptoms compared to those without such experiences (AOR = 14.46; 95 % CI: 5.75–36.34). Likewise, mothers with a history of depression exhibited nearly threefold higher odds of postpartum depressive symptoms (AOR = 3.11; 95 % CI: 1.36–7.08). Social support also demonstrated a strong gradient effect: mothers reporting low support had almost thirteen times greater odds (AOR = 12.72; 95 % CI: 3.77–42.90), while those with medium support had approximately threefold greater odds (AOR = 2.67; 95 % CI: 1.28–5.60) compared with mothers who reported high support (Table 3).

### Obstetric and infant-related factors associated with PPD symptoms

In the bivariate analyses, parity, abortion history, gestational age, pregnancy complications, childbirth complications, availability of postpartum assistance, and infant sleep difficulties were significantly associated with postpartum depressive symptoms. However, after controlling for potential confounders in the multivariable logistic regression model, only pregnancy complications and infant sleep difficulties remained significant independent correlates of postpartum depressive symptoms (p < 0.05).

Specifically, mothers who experienced pregnancy complications had more than twice the odds of postpartum depressive symptoms compared with those without complications (AOR = 2.55; 95 % CI: 1.12–5.82). Similarly, mothers whose infants experienced sleep difficulties had more than double the odds of depressive symptoms relative to mothers whose infants slept well (AOR = 2.53; 95 % CI: 1.27–5.05) (Table 4).

The Hosmer–Lemeshow goodness-of-fit test indicated adequate model fit for Table 3 (χ² = 10.16, p = 0.18) and Table 4 (χ² = 11.30, p = 0.13), suggesting that the models fit the data well.

**Table 3:** Bivariate and multivariate logistic regression of postpartum depressive symptoms and associated socio-demographic and social support factors among postpartum mothers in Mogadishu, Somalia, 2024 (n = 271).

**Variable**	**Category**	**PPD symptoms**		**COR(95 %CI)**	**AOR(95 %CI)**	**P-vale**
		**Yes (%)**	**No (%)**			
Education status	Non-formal education	25 (37.9)	41 (62.1)	1.78(.920–3.46)	1.84(.838–4.02)	.129
	Primary school	8 (25)	24 (75)	.975(.392–2.43)	1.26(.449–3.52)	.662
	Secondary school	12 (17.9)	55 (82.1)	.638(.298–1.37)	.663(.260–1.69)	.389
	Degree and above	27 (25.5)	79 (74.5)	1	1	
Stressful live events	Yes	32(78)	9(22)	16.89(7.48–38.13)	14.46(5.75–36.34)	< .001*
	No	40(17.4)	190(82.6)	1	1	
Presence of chronic illness	Yes	15 (50)	15 (50)	3.23(1.49–7.01)	1.79(.687–4.67)	.233
	No	57 (23.7)	184 (76.3)	1	1	.989
History of depression	Yes	33 (60)	22 (40)	6.81(3.59–12.93)	3.11(1.36–7.08)	.007*
	No	39(18.1)	177(81.9)	1	1	
Social support	Low support	7(35)	13(65)	8.54(3.09–23.59)	12.72(3.77–42.90)	< .001*
	Medium support	77(69.4)	34(30.6)	2.03(1.12–3.67)	2.67(1.28–5.60)	.009*
	High support	115(82.1)	25(17.9)	1	1	

* indicates significance at 5 % level, PPD: Postpartum depression, COR: Crude odd ratio, AOR: Adjusted odd ratio 1: reference categories, CI: Confidence interval.

**Table 4:** Bivariate and multivariate logistic regression of postpartum depressive symptoms and associated obstetric and baby-related factors among postpartum mothers in Mogadishu, Somalia 2024 (n = 271).

**Variable**	**Category**	**PPD symptoms**		**COR (95 %CI)**	**AOR (95 %CI)**	**P-vale**
		**Yes (%)**	**No (%)**			
Number of parities	1	18 (34.6)	34 (65.4)	1	1	
	2–4	26 (21.5)	95 (78.5)	0.517(.252–1.06)	.763(.339–1.72)	.515
	Above 4	28 (28.6)	70 (71.4)	.756(.368–1.55)	.952(.412–2.20)	.909
History of abortion	Yes	40 (33.3)	80 (66.7)	1.86(1.08–3.21)	1.63(.926–2.85)	. 091
	No	32 (21.2)	119 (78.8)	1	1	
Last pregnancy gestational age	Preterm	13 (68.4)	6 (31.6)	3.23(.776–13.45)	.938 (.320–2.75)	.907
	Full term	158 (71.8)	62 (28.2)	2.75(.925–8.15)	.356(.078–1.634)	.184
	Post term	28 (87.5)	4 (12.5)	1	1	
Complications during pregnancy	Yes	13 (42.3)	15 (57.7)	2.70(1.22–6.01)	2.55(1.12–5.82)	.026*
	No	59 (24.3)	184 (75.7)	1	1	
Complications at childbirth	Yes	7 (53.8)	6 (46.2)	3.46(1.12–10.68)	1.59(.406–6.201)	.507
	No	65 (25.2)	193 (74.8)	1	1	
Availability of assistance	Yes	28 (32.6)	58 (67.44)	1.55(.880–2.72)	1.28(.702–2.35)	417
	No	44 (23.8)	141 (76.2)	1	1	
Problems with baby sleeping	Yes	20 (45.5)	24 (54.5)	2.80(1.44–5.48)	2.53(1.27–5.05)	.008*
	No	52 (22.9)	175 (77.1)	1	1	

* indicates significance at 5 % level, PPD: Postpartum depression, COR: Crude odd ratio, AOR: Adjusted odd ratio 1: reference categories, CI: Confidence interval.

## Discussion

This study aimed to assess the prevalence of PPD symptoms and identify associated factors among postpartum mothers in Mogadishu, Somalia. The primary factors significantly associated with PPD symptoms included stressful life events, history of depression, infant sleep difficulties, complications during pregnancy, and low to medium levels of social support. Importantly, the study found social support to act as a protective factor against developing postpartum depression.

The observed prevalence of postpartum depressive symptoms in this study was 26.6 % (95 % CI: 21.4– 32.2 %), slightly higher than the 20 % previously reported in Somalia [16], yet substantially lower than the 69.9 % prevalence reported by another local study [15]. This prevalence aligns closely with findings from studies conducted in Ethiopia [23], [24], Uganda [25], Senegal [26], and Syria [27]. Additionally, our findings are consistent with a recent systematic review and meta-analysis from the Middle East, which reported a pooled prevalence similar to the current results [28]. Furthermore, an umbrella review encompassing 45 countries across five continents documented a global PPD prevalence rate in line with our findings [29]. However, the prevalence rate in the present study was notably lower than that reported in certain studies from Ethiopia [9], [30], the United Arab Emirates (UAE) [31], Kazakhstan [32], Saudi Arabia [33], and Palestine [34]. Conversely, our reported prevalence is higher compared to other studies conducted among postpartum mothers in Ethiopia [35], Eritrea [36], Kenya [11], Turkey [37], and China [38].

Variations in sample size, the depression assessment tools utilized, chosen cut-off scores, study designs, and socio-cultural disparities between Somalia and other regions likely contributed to the observed differences in PPD prevalence rates. For instance, studies from Eritrea [36] and Uganda [25] applied DSM-5 diagnostic criteria, whereas many others utilized the Edinburgh Postnatal Depression Scale (EPDS) [15], often with varying cut-off points [9], [23], [24], [30], [35], [38]. Furthermore, differences in timing of assessments within the postpartum period may significantly influence the detection rates of depressive symptoms. Supporting this, a recent longitudinal study, which collected data at three distinct intervals throughout the first postpartum year, demonstrated considerable variability in symptom prevalence across these time points [39]. Similarly, findings from another study conducted in Iran align with these observations, underscoring the importance of timing in postpartum depression evaluations [40]. Additional factors potentially contributing to discrepancies include individual differences in literacy and test-taking abilities, variations in symptom-reporting behaviors, cultural perceptions of mental health, stigma associated with psychiatric disorders, and biological vulnerability to PPD [36], [41]. Systematic reviews and meta-analyses also report varying prevalence rates. Two recent systematic reviews, one conducted in India and another covering Sub-Saharan Africa, each reported a prevalence of approximately 22 % [42], [43], slightly lower than the present study. Furthermore, a comprehensive systematic review encompassing 565 studies across 80 countries documented a global pooled prevalence of 17.22 % [44], also lower than our observed prevalence. These findings reinforce the necessity of contextualizing postpartum depression within regional, methodological, and cultural frameworks.

Pregnancy is a complex and often stressful phase of life. An effective social support network from partners, family members, and friends during the postpartum period is essential for alleviating pregnancy-related stress and mitigating challenges associated with motherhood. In contrast, insufficient or absent social support may exacerbate feelings of isolation, exhaustion, and stress, significantly increasing the likelihood of developing PPD symptoms. Robust social support systems can offer emotional comfort, practical assistance, and validation of maternal experiences, thereby acting as protective buffers against depressive symptoms. In this study, mothers who perceived low or moderate levels of social support demonstrated significantly higher odds of experiencing PPD symptoms compared to those with high social support, consistent with previous findings from Uganda [25], Ethiopia [10], [35], [45], [46], and Palestine [34]. A recent systematic review and meta-analysis comprising 13 studies similarly found that postpartum mothers lacking adequate social support were approximately six times more likely to develop depressive symptoms compared to those who received sufficient social support [47]. Conversely, previous studies conducted in Mogadishu, Somalia [16], and Dessie, Ethiopia [24], did not find comparable associations between social support and PPD symptoms. Furthermore, different sources and types of social support may exert varying influences on the likelihood of developing postpartum depression. According to Yamada et al. [48], mothers who lack support from their partners but receive support solely from parents, relatives, or friends exhibited significantly higher odds of postpartum depression compared to those receiving support from both partners and family members. This suggests the importance of partner involvement as a particularly critical component of social support networks.

Stressful life events experienced during pregnancy emerged as another significant factor associated with PPD symptoms in this study. Such events typically include frequent arguments with a partner, separation or divorce, physical altercations, relocation, financial difficulties, unemployment, or illness and death among close family members [49]. Existing research highlights that stress can elevate amygdala activity, potentially leading to mood disturbances and an increased risk of depression [50]. Correspondingly, our study found that mothers who reported stressful events during pregnancy had approximately 11.6 times higher odds of experiencing PPD symptoms than those without such events. This aligns with findings from Ethiopia [51], Senegal [26], Palestine [34], and Saudi Arabia [33]. A related UK study also found that relationship-related stressors occurring during pregnancy and postpartum increased women's vulnerability to developing depressive symptoms, especially when such events preceded depressive episodes by a few months [52]. However, a prior study conducted in Mogadishu, Somalia, did not find similar results, possibly reflecting methodological or contextual differences [16].

It is well-established that a previous depressive episode strongly predicts subsequent episodes. Consistent with this, our study demonstrated that mothers with a history of depression had significantly higher odds of experiencing postpartum depressive symptoms compared to those without prior depressive episodes. These findings corroborate previous studies highlighting the vulnerability conferred by a history of depression to developing postpartum depressive symptoms [10], [38], [53], [54], [55], further supported by a recent systematic review and meta-analysis conducted in Ethiopia [56]. This underscores the necessity for healthcare professionals to proactively identify and support mothers with prior depressive episodes due to their elevated risk. Nonetheless, contrasting findings have been reported from studies conducted in Egypt [56], Ethiopia [9], [23], [46], [51], and Saudi Arabia [33], suggesting potential variations related to demographic, socio-cultural, or methodological factors.

Complications during pregnancy, such as pre-eclampsia, gestational diabetes, fetal distress, maternal- fetal infections, and hemorrhage, are also associated with heightened risks of adverse psychosocial outcomes, including postpartum depression [57]. In this study, mothers who reported complications during pregnancy exhibited significantly higher prevalence rates of postpartum depressive symptoms compared to those without complications. This finding is consistent with earlier reports from Ethiopia [45], Kazakhstan [32], and a recent systematic review and meta-analysis conducted in the Middle East, which demonstrated increased odds of developing PPD among mothers experiencing pregnancy complications [28]. Conversely, other studies from Kenya [11], Uganda [25], Nigeria [58], and Ethiopia [34], [54] did not find associations between pregnancy complications and PPD, potentially due to differences in population characteristics, assessment methods, or sample sizes.

Sleep constitutes approximately one-third of human life and plays an essential role in cognitive

functioning and emotional well-being. A strong reciprocal relationship exists between sleep disturbances and depression. While disturbed sleep is commonly considered a symptom of depression, evidence indicates that disrupted or insufficient sleep may also directly contribute to the development of mood disorders [59]. In this study, mothers whose infants experienced sleep difficulties had approximately three times greater odds of experiencing PPD symptoms compared to mothers whose infants slept normally. These findings are consistent with previous studies conducted in Ethiopia [10], Cameroon [60], and Egypt [61]. One plausible explanation is that infant sleep problems during early motherhood can lead to maternal sleep deprivation, exhaustion, and subsequent depressive symptoms. However, contrasting findings were reported by a study conducted in Uganda, which found no significant association between infant sleep disturbances and postpartum depressive symptoms [25].

In this study, a history of abortion, unplanned pregnancy, or chronic medical conditions was not significantly associated with postpartum depressive symptoms. This aligns with findings from earlier studies which similarly reported no significant associations between postpartum depression and abortion history [62], unintended pregnancies [45], or chronic medical conditions [25]. Nevertheless, divergent results have been documented in other studies; for instance, Asaye et al. [23] identified a significant link between abortion history and PPD symptoms, Gebregziabher et al. [36] reported associations with unplanned pregnancy, and Abebe et al. [51] found an increased risk among mothers with chronic medical conditions. These discrepancies may reflect differences in study designs, participant characteristics, sample sizes, or sociocultural contexts.

These findings should be interpreted within the sociocultural context of Somalia, where mental health remains highly stigmatized and access to professional psychiatric care is extremely limited [63]. Cultural and religious expectations that emphasize maternal resilience, strength, and self-sacrifice may discourage women from acknowledging or disclosing emotional distress, leading to underreporting of symptoms. In addition, reliance on family networks and faith-based coping, coupled with the limited availability of formal psychosocial services, may significantly shape both the experience and management of postpartum depression.

## Limitations

This study has several limitations. First, its cross-sectional design precludes establishing cause-and-effect relationships. For example, it is unclear whether inadequate social support increases depressive symptoms or if depressive symptoms reduce the perception or availability of support. Future longitudinal studies should clarify these causal pathways. Second, reliance on self-report questionnaires rather than clinician-administered diagnostic assessments introduces potential recall and reporting biases. Third, depressive symptoms were assessed using the EPDS, a screening tool rather than the gold-standard DSM-5 diagnostic criteria. Finally, although the EPDS and the MSSS are widely validated internationally, they have not undergone formal validation in the Somali cultural and linguistic context. This may limit cultural validity and interpretation of results. Future research should prioritize adaptation and validation of these psychometric instruments for the Somali setting.

## Conclusion and recommendations

This study found that postpartum depression affects a substantial proportion of mothers (26.6 %) in Mogadishu, Somalia. Key factors associated with PPD symptoms included stressful life events, a prior history of depression, infant sleep difficulties, pregnancy complications, and low or moderate social support.

Given the high prevalence, urgent measures are needed to integrate routine PPD screening, psychological interventions, and psychoeducational programs into maternal health services. Healthcare professionals should be trained to recognize and address depression risk factors, particularly those common in Somalia. Regular screening combined with timely interventions and follow-up care would help prevent chronicity and relapse.

In addition, perinatal care programs should incorporate education on postpartum challenges, coping strategies, and early recognition of depressive symptoms, empowering women to seek help proactively. Finally, national health policies should formally address postpartum depression, with emphasis on strengthening referral systems, expanding access to psychosocial services, and raising awareness among policymakers, providers, and communities. Such initiatives are critical to safeguarding maternal well-being and fostering healthier mother–child relationships.

## Ethics approval and consent to participate

This study was approved by the hospital’s ethics review board (MSTH/15320). All methods were carried out in accordance with the Declaration of Helsinki. Written informed consent was obtained from all participants.

## CRediT authorship contribution statement

Rahma Yusuf Haji Mohamud and Nur Adam Mohamed were the study's principal investigators and was involved from inception to design, data acquisition, analysis and interpretation, and manuscript drafting and editing. Yusuf Abdirisak Mohamed, Khadija Yusuf Ali, Nur Adam Mohamed, Said Mohamed Mohamud, Serpil Doğan, Nazan Karahan, Said Abdirahman Ahmed, Alia Ismail Hashi, IMO, AIH, Zerife Orhan and Mohamed Yaqub Hassan were involved in the review of the proposal, tool evaluation, interpretation, data collection, and critical review of the draft manuscript. All authors read and approved the final manuscript**.**

## Funding

This study received no specific funding from funding agencies or sectors.

## Declaration of Competing Interest

The authors declare that they have no competing interests.

## Data Availability

All data generated or analyzed during this study are included in this published article or are available from the corresponding author upon reasonable request.
